# The transgenerational inheritance of autism-like phenotypes in mice exposed to valproic acid during pregnancy

**DOI:** 10.1038/srep36250

**Published:** 2016-11-07

**Authors:** Chang Soon Choi, Edson Luck Gonzales, Ki Chan Kim, Sung Min Yang, Ji-Woon Kim, Darine Froy Mabunga, Jae Hoon Cheong, Seol-Heui Han, Geon Ho Bahn, Chan Young Shin

**Affiliations:** 1 Department of Neuroscience, School of Medicine and Neuroscience Research Center, Institute SMART-IABS, Konkuk University, Seoul 143-701, Korea; 2 Department of Pharmacy, Sahmyook University, Seoul, Korea; 3 Department of Neuropsychiatry, School of Medicine, Kyung Hee University, Seoul, Korea

## Abstract

Autism spectrum disorder (ASD) is a heterogeneously pervasive developmental disorder in which various genetic and environmental factors are believed to underlie its development. Recently, epigenetics has been suggested as a novel concept for ASD aetiology with a proposition that epigenetic marks can be transgenerationally inherited. Based on this assumption of epigenetics, we investigated the transgenerational inheritance of ASD-like behaviours and their related synaptic changes in the VPA animal model of ASD. The first generation (F1) VPA-exposed offspring exhibited autistic-like impaired sociability and increased marble burying. They also showed increased seizure susceptibility, hyperactivity and decreased anxiety. We mated the VPA-exposed F1 male offspring with naïve females to produce the second generation (F2), and then similarly mated the F2 to deliver the third generation (F3). Remarkably, the autism-like behavioural phenotypes found in F1 persisted to the F2 and F3. Additionally, the frontal cortices of F1 and F3 showed some imbalanced expressions of excitatory/inhibitory synaptic markers, suggesting a transgenerational epigenetic inheritance. These results open the idea that E/I imbalance and ASD-like behavioural changes induced by environmental insults in mice can be epigenetically transmitted, at least, to the third generation. This study could help explain the unprecedented increase in ASD prevalence.

Autism spectrum disorder (ASD) is a heritable and heterogeneous neurodevelopmental disability mainly manifested by defects in social communication and repetitive behaviors usually detected before the age of 3 years^1^. The prevalence rate of ASD in the United States is about 1 in 68 children in 2010, more than a hundred-fold increase since 2000^2^. It is thus paramount to understand the reasons for the increased prevalence of ASD as it has become a major challenge in the field due to its heterogeneity resulting to lacks of unifying etiology and pathophysiology, and the scarcity of treatment options. Moreover, the families of ASD patients are greatly affected throughout life having economic burdens, social stigma and the high risk of passing ASD susceptibility genes to the next generation.

The most commonly suggested etiology of ASD is through the hereditary genetic characteristics identified as high risk genes for ASD and can be aggravated by prenatal injuries, infection and other environmental insults^3^. Previous studies found that the ASD-related concordance rates in monozygotic twins of the European populations are as high as 60 to 90%, topping all other heritable developmental disorders^3^,^4^,^5^,^6^. However, later studies of larger sample populations refuted the previous findings of ASD high concordance rates as overestimated since about half of ASD cases can be due to intriguingly either shared environmental^7^ or non-shared environmental factors^8^ between twins or the normal population. Thus, exposure to environmental factors imposes a significant contribution to ASD development in the prenatal and early postnatal periods. The epigenetic mechanism is demonstrated as the relative change in gene activity, either repress or activate, without altering its DNA sequence in a mature cell through alterations in chromatin structure by histone modification and DNA methylation^9^,^10^. Consequently, epigenetic changes induced by environmental factors, such as long-term drug use in the case of addiction, can drive permanent changes in cellular and synaptic structures that shape behavioral phenotypes^11^. One interesting assumption of the remodeled epigenetic makeup is that it opens the possibility of increased susceptibility of transmitting new traits to the subsequent generations, thus the term “transgenerational epigenetic inheritance”^12^. Simply put, the marked changes on DNA to alter gene expression can be acquired and passed from parent to offspring through the sperm or eggs, thereby affecting the phenotypes of the subsequent generation of offspring.

When the DNA’s epigenetic state and its associated phenotypes are inherited to the third generation offspring (F3) after toxicant exposure was induced in the first pregnant mother (F0), transgenerational epigenetic inheritance occurs^13^. Epigenetic inheritance can be widely influenced by the environment such as diet, as what has been elucidated in the *agouti viable yellow (A*^*vy*^) mice^14^, and chemical exposures during pregnancy as elucidated in medaka fish^15^. Other parental conditions such as lifetime stress can change the epigenetic marks in the germ cells^16^. Bertram and his colleagues also reported that maternal undernutrition in guinea pigs caused dramatic changes in heart structure and hypothalamic-pituitary-adrenal (HPA) function across two generations^17^. This concept can have important implications in the environmental factors affecting pregnancy outcome and causing neurodevelopmental disorders in the offspring such as ASD. A well-known environmental risk factor of ASD in humans is the valproic acid (VPA), an anti-epileptic drug with an identified histone deacetylase inhibitor property^18^. The VPA-exposed animals have become one of the most widely used models of ASD for its clinical relevance^19^,^20^,^21^,^22^. Given the effects of VPA in the epigenetic characteristics of developing animals, we hypothesize that VPA treatment can induce a transgenerational epigenetic inheritance of ASD phenotypes and synaptic changes across multiple generations at least up to F3. Using the VPA animal model, we investigated the transmission of autistic phenotypes across three generations of mice to validate our hypothesis. The outcome of this study will provide insights into the contributing factors of increased ASD prevalence over the years.

## Results

### Transgenerational effect of VPA exposure on body weights and birth rate

**Figure 1:**
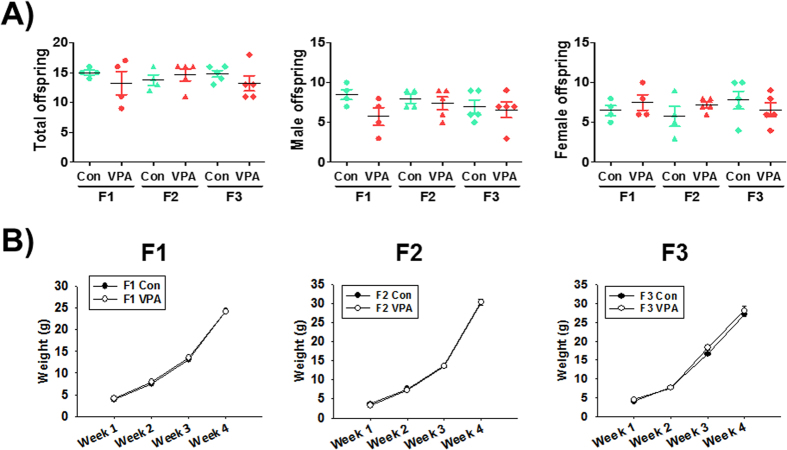
Transgenerational effect of VPA in litter size and body weights of exposed mice. The number of live pups in each litter and in every generation were measured at postnatal day 7. Body weights of control and VPA-exposed mice in every generation were measured from postnatal day 7 to 28. Pups number (**A**) and body weights (**B**) were not significantly different between the control and VPA-exposed group for each generation. All data are expressed as the mean ± S.E.M. (n = 4–5 litters per group and generation).

We checked the number of live births (total and according to sex) from each dam in each generation. The total number of live births in each generation did not differ significantly between control and VPA-exposed mice (Fig. 1A). There was also no difference in the number of animals between F1 to F3 generations. To investigate the effect of prenatal VPA exposure on the body weight profile of pups in each generation, we checked their weight for 3 weeks between postnatal day 7 and 28. There was no significant difference between the control group and the VPA-exposed group (Fig. 1B). These results suggested that VPA does not affect the reproductive capacity as well as the viability of mice offspring.

### Transgenerational effect of VPA on crooked tail phenotype

**Figure 2:**
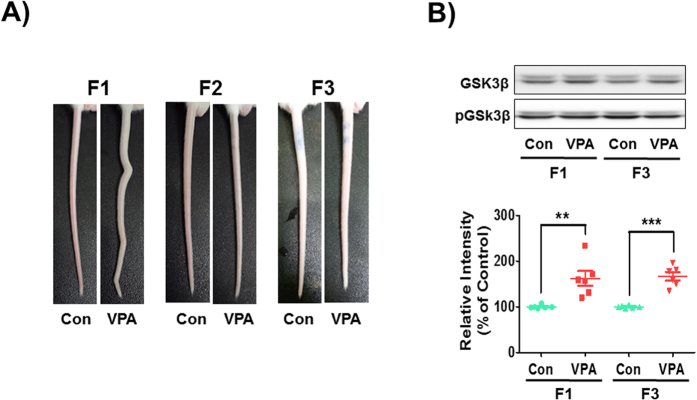
Neural tube defects by VPA exposure and its transgenerational effect. (**A**) Malformations of tail structure occurred in F1 VPA-exposed mice, but not in the F2 and F3. (**B**) Protein levels of phosphorylated-GSK3β were examined using Western blot in F1 and F3 generation mice frontal cortex at embryonic day 14. All data are expressed as the mean ± S.E.M. (n = 12 mice randomly selected from 6 litters per group and per generation). **p < *0.01*, ***p < *0.001* vs. control group as revealed by post-hoc Bonferroni’s comparisons.

Neural tube defects (NTD) can result from genetic mutation, malnutrition or exposure to teratogens during gestation^23^. VPA is a known inducer of NTD and causes crooked tail phenotypes, a mild form of NTD, and autistic-like behavioral abnormalities in mice and rats^24^,^25^. It was shown that crooked-tail phenotype occurs in the VPA-exposed F1 group^26^. However, the subsequent F2 and F3 generations did not have crooked tails suggesting no transgenerational transmission of skeletal defects (Fig. 2A).

Glycogen synthase kinase 3 beta (GSK3β), which is regulated by Wnt/β-catenin signaling pathway, has an essential role in the regulation of neurogenesis wherein its overexpression is correlated with macrocephaly and VPA-induced ASD phenotypes^27^,^28^. We isolated the neocortex in F1 and F3 mice fetus at embryonic day 14 and examined the expression of phosphorylated GSK3β (pGSK3β). Although the crooked tail phenotype was not transmitted through F3 generation in VPA-exposed groups, the pGSK3β expressions were maintained at high levels in both F1 and F3 VPA-exposed groups [F1: t_(10)_ = 3.790, p < 0.0035; F3: t_(10)_ = 7.139, p < 0.0001] (Fig. 2B). These results suggested that VPA alteration of GSK3β activation follow through the F3 generation and the crooked tail phenotype could be caused by the direct teratogenic effect of VPA other than the changes in epigenetics.

### Transgenerational effect of VPA in autism-related core symptoms

**Figure 3:**
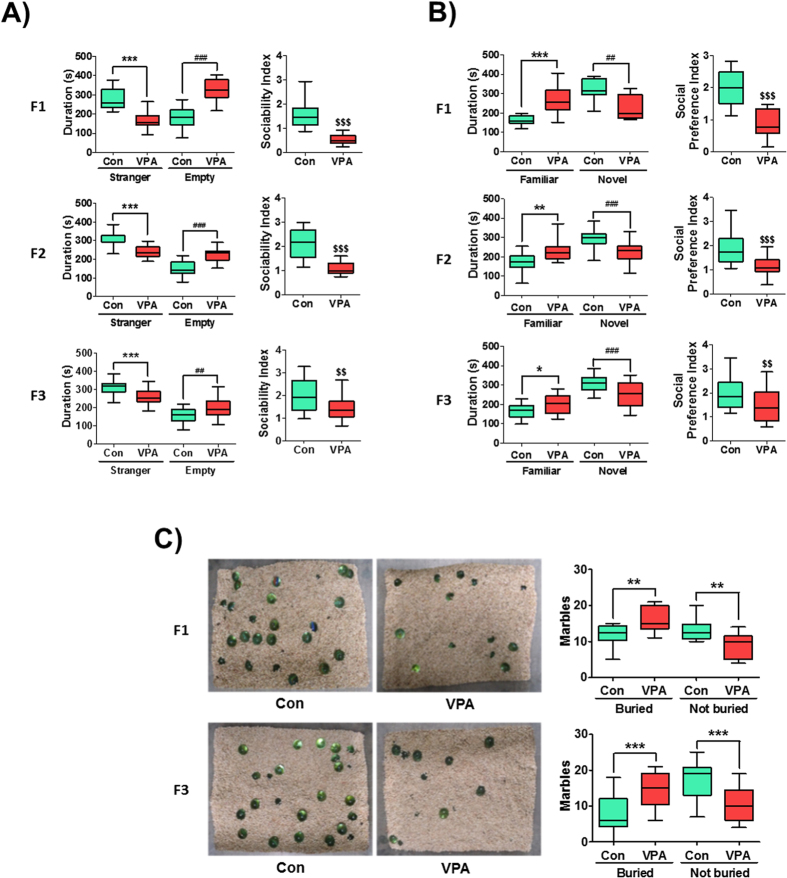
Transgenerational effect of ASD-like behaviors in VPA-exposed mice. (**A**) VPA exposure reduced the sociability of F1, F2 and F3 mice. The duration of stay (left panel) in stranger 1 (S1) side and empty side was shown in the graph. Sociability index (SI, right panel) was calculated as the ratio of time spent in stranger 1 side over empty side. ***p < *0.001* vs. control groups in S1 side; ^##^*p* < *0.01*, ^###^*p* < *0.001* vs. control groups in empty side; ^$$^*p* < *0.01*, ^$$$^*p < 0.001* vs. control groups in SI as revealed by post-hoc Bonferroni’s comparisons (F1: n = 15 for Con and 17 for VPA; F2: n = 15 for Con and 20 for VPA; F3: n = 29 for Con and 20 for VPA; all mice were randomly selected from 6 litters per group and per generation). (**B**) VPA exposure reduced the social preference in F1, F2 and F3 mice. The duration of stay in familiar side (stranger 1, S1) and novel side (stranger 2, S2) was shown in the graph. Social preference index (SPI, right panel) was calculated as the ratio of time spent in S2 side over S1 side. *p < *0.05,* **p < *0.01,* ***p < *0.001* vs. control group in S1 side; ^##^*p < 0.01*, ^###^*p* < *0.001* vs. control groups in S2 side; ^$$^*p* < *0.01*, ^$$$^*p* < *0.001* vs. control group in SPI as revealed by post-hoc Bonferroni’s comparisons. The animals used for this experiment is the same with the sociability test. (**C**) VPA exposure increased the repetitive behaviors in F1 and F3 mice as determined by the marble burying test. **p < *0.01,* ***p < 0.001 vs. control group in each generation as revealed by post-hoc Bonferroni’s comparisons. (F1: n = 10 for Con and 9 for VPA; F3: n = 20 for Con and 20 for VPA; all mice were randomly selected from 6 litters per group and per generation).

VPA injection during embryonic day 12 (E12) gave consistent and robust changes in the social interaction of rodent offspring^26^. In this study, we investigated whether or not VPA exposure induces transgenerational effects of ASD-related behaviors. The sociability of mice was determined at 4 weeks of age. In the VPA-exposed F1 group, the duration in stranger chamber was lower [t_(30)_ = 6.94, p < 0.0001] while in the empty side was higher [t_(30)_ = 7.68, p < 0.0001] (Fig. 3A) compared to the F1 control group. The sociability index (SI) was determined as the quotient between stranger side divided by the empty side durations. The SI was significantly decreased in the F1 VPA group compared with F1 control group [t_(30)_ = 7.02, p < 0.0001] (Fig. 2A). Interestingly, the VPA-exposed F2 and F3 generations had similar impairments of sociability in the stranger chamber [F2: t_(33)_ = 6.03, p < 0.0001; F3: t_(47)_ = 3.18, p = 0.0026] with an increased time spent in the empty chamber [F2: t_(33)_ = 6.27, p < 0.0001; F3: t_(47)_ = 4.42, p < 0.0001] compared to their respective controls. The SI of VPA-exposed groups in the F2 and F3 generations were affirmably lower than their respective controls [F2: t_(33)_ = 5.75, p < 0.0001; F3: t_(47)_ = 3.07, p = 0.0034] (Fig. 3A).

In the subsequent test of preference for social novelty, the F1 VPA-exposed mice stayed more time in the familiar chamber [t_(30)_ = 4.35, p = 0.0003] and less time in the novel chamber [t_(30)_ = 3.74, p = 0.0014] compared with F1 control. The social preference index (SPI) was calculated as the quotient of time spent in the novel divided by the familiar chambers. As a result, the SPI of VPA-exposed F1 was significantly lower than the F1 control [t_(30)_ = 5.38, p < 0.0001]. In the F2 and F3 generations, VPA-exposed mice also had significantly increased time spent in the familiar compartment [F2: t_(33)_ = 3.43, p = 0.0015; F3: t_(47)_ = 2.62, p = 0.012] and decreased in the novel compartment [F2:: t_(33)_ = 4.17, p = 0.0002; F3: t_(47)_ = 3.76, p = 0.0005] compared to their respective controls. The SPI of VPA-exposed F2 and F3 generations were significantly lower than their respective controls [F2: t_(33)_ = 4.39, p < 0.0001; F3: t_(47)_ = 2.98, p = 0.0045].

To investigate the repetitive behavioral phenotype, we performed the marble burying test. The VPA-exposed F1 mice buried more marbles than the control group [t_(17)_ = 2.92, p = 0.0096], in which this behavior was also observed in the VPA-exposed F3 generation [t_(38)_ = 4.07, p = 0.0002] (Fig. 3C).

### Transgenerational effect of VPA in autism related comorbid symptoms

**Figure 4:**
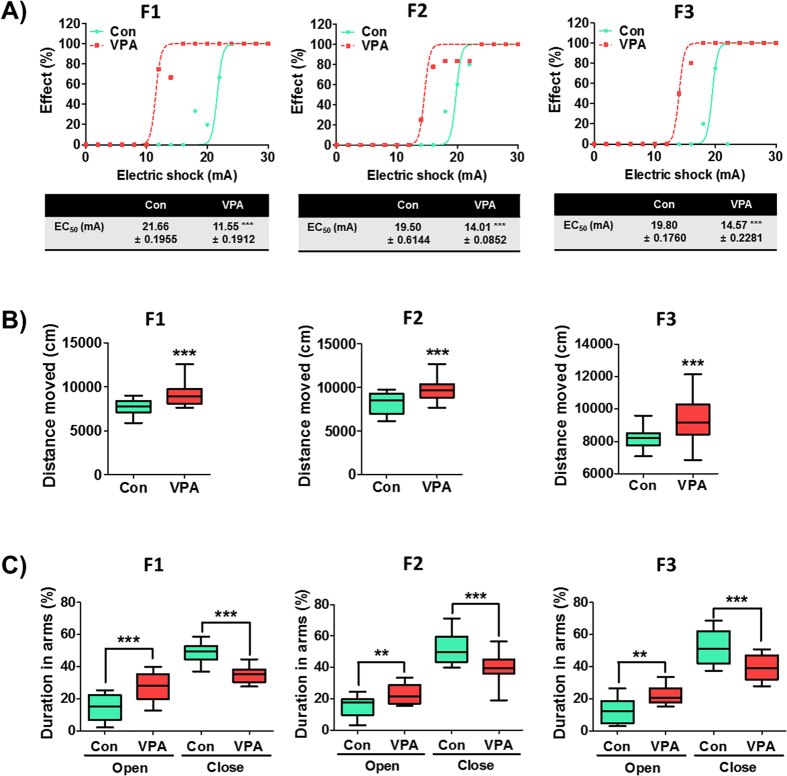
Prenatal VPA exposure induces a transgenerational effect on accompanying symptoms of ASD. (**A**) Increased sensitivity to electroshock stimulation in F1, F2 and F3 VPA-exposed mice. The CC50 of VPA groups in each generation were significantly lower than that of their respective controls (***p < 0.001). The tables show the actual values of CC50 with upper and lower confidence limits. (F1: n = 14 for Con and 12 for VPA; F2: n = 12 for Con and 17 for VPA; F3: n = 20 for Con and 30 for VPA; all mice were randomly selected from 6 litters per group and per generation). (**B**) Hyperactivity of VPA-exposed mice in F1, F2 and F3 generations determined by the open field test. The total distance moved was significantly increased in VPA groups of each generation. (F1: n = 29 for Con and 32 for VPA; F2: n = 24 for Con and 22 for VPA; F3: n = 34 for Con and 65 for VPA; all mice were randomly selected from 6 litters per group and per generation). (**C**) The anti-anxiety behaviors of VPA-exposed mice in each generation were investigated in the elevated plus maze. The percentages of time spent in the open arms were significantly increased in the VPA groups of each generation. (F1: n = 13 for Con and 10 for VPA; F2: n = 19 for Con and 14 for VPA; F3: n = 10 for Con and 15 for VPA; all mice were randomly selected from 6 litters per group and per generation). All data are expressed using box plot diagram. **p < *0.01,* ***p < *0.001* vs. control group of each generation as revealed by post-hoc Bonferroni’s comparisons.

Increased excitatory tone affects the excitability of neurons, which is often increased in the ASD brain. In the electroshock-induced seizure, the VPA-exposed F1 have significantly lower seizure threshold than F1 control (p < 0.001). This phenomenon was also observed in the F2 and F3 generations of the VPA-exposed F1 mice (p < 0.001) (Fig. 4A).

Clinically, some ASD patients have inattention and hyperactivity^29^,^30^. Accordingly, hyperactivity behaviors were also observed in the VPA-exposed animal models^31^. We then investigated whether VPA induces a transgenerational effect on the hyperactivity phenotype (Fig. 4B). Indeed, VPA-exposed F1 mice displayed significantly greater locomotor activity than F1 control as determined by the total distance moved in the open field [t_(59)_ = 5.05, p < 0.0001]. This behavior also persisted to the F2 and F3 generations wherein they have significantly higher total distance moved than controls [F2: t_(44)_ = 4.39, p < 0.0001; F3: t_(97)_ = 5.19, p < 0.0001]. We checked the distance moved in the center area as an indicator of aggression or lack of anxiety (impulsivity). The result revealed that VPA-exposed F1 mice have more activity in the center area than control mice, which were also true in the F2 and F3 generations (data not shown).

We conducted the elevated plus maze (EPM) test to confirm whether increased duration in the center area of the open field test is related to decreased anxiety in this paradigm (Fig. 4C). Indeed, the VPA-exposed F1 mice had more time spent than controls in the open arms [t_(21)_ = 3.83, p = 0.001] and vice versa in the close arms [t_(21)_ = 4.48, p = 0.0003]. More interestingly, the F2 and F3 generations of the VPA-exposed group displayed a persistent lack of anxiety than their respective controls [F2: t_(31)_ = 2.99, p value = 0.0055 in the open arm; t_(31)_ = 3.81, p value = 0.0006 in the close arm; F3: t_(23)_ = 3.69, p value = 0.0012 in the open arm; t_(23)_ = 3.79, p value = 0.0008 in the close arm].

### Transgenerational induction of neuronal abnormalities in the F0 VPA-exposed generations

**Figure 5:**
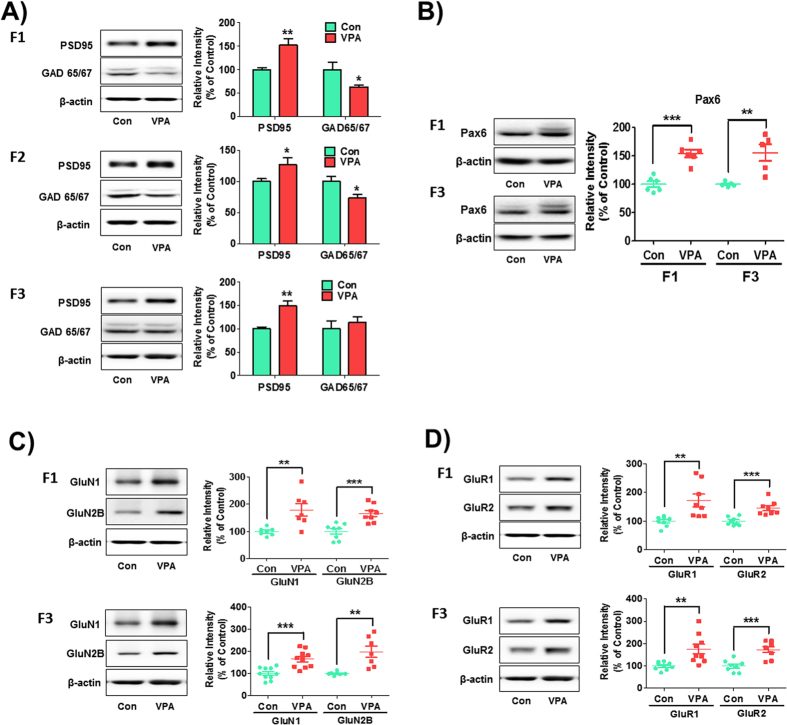
Increased expression of excitatory postsynaptic proteins in F1, F2 and F3 VPA-exposed mice. Western blot analysis was performed using brain lysates from control and VPA-exposed mice of each generation at 4 weeks (**A**) for PSD95 [F1: n = 6 for Con and 5 for VPA; F2: n = 14 per group; F3: n = 5 for Con and 6 for VPA; all were randomly selected from 5–6 litters per group and per generation], and GAD 65/67 [F1: n = 8 per group; F2: n = 29 for Con and 24 for VPA; F3: n = 5 for Con and 6 for VPA; all were randomly selected from 5–6 litters per group and per generation]) and at E14 (**B**) for Pax6 [F1: n = 6 per group; F3: n = 5 per group; all were randomly selected from 5–6 litters per group and per generation]). Protein levels of NMDA (**C**) and AMPA (**D**) receptors were examined in the frontal cortex of F1 and F3 VPA-exposed mice offspring at postnatal week 4. To analyze the abnormalities in NMDA receptors, the protein levels of GluN1 (F1: n = 7 per group; F3: n = 10 per group; all were randomly selected from 6 litters per group and per generation) and GluN2B (F1: n = 8 per group; F3: n = 7 per group; all were randomly selected from 6 litters per group and per generation) were examined by Western blot. For AMPA receptors, the protein levels of GluR1 (F1: n = 8 per group; F3: n = 9 per group; all were randomly selected from 6 litters per group and per generation) and GluR2 (F1: n = 8 per group; F3: n = 8 per group; all were randomly selected from 6 litters per group and per generation) were examined. Cropped representative blots were shown for each protein of interest along with quantitative graphs of image analyses calculated as the percentage of expression relative to control groups normalized as 100%. *p < *0.05,* **p < *0.01,* ***p < *0.001* vs. control group as revealed by post-hoc Bonferroni’s comparisons.

As previously reported, VPA induced abnormal neuronal differentiation with increased excitatory neuronal marker expression such as PSD-95 and vGluT1 and decreased inhibitory neuronal marker expression such as GAD in exposed offspring^32^. To evaluate whether the abnormal neuronal differentiation induced by VPA could have transgenerational inheritance, we measured the expression of post-synaptic markers PSD-95 and the inhibitory neuronal marker GAD 65/67, in the cortex of each generation (Fig. 5A). In the F1 generation, the expression of PSD-95 was significantly increased in the VPA-exposed group [t_(9)_ = 3.76, p value = 0.0043]. Interestingly, the PSD-95 band intensities of VPA-exposed F2 and F3 mice were also higher than their respective controls [F2: t_(26)_ = 2.10, p value = 0.045; F3: t_(9)_ = 3.81, p value = 0.0041]. In case of GAD 65/67, the expression was significantly reduced in the VPA-exposed F1 [t_(16)_ = 2.20, p value = 0.035] and F2 generations [t_(51)_ = 2.41, p value = 0.020] but not in F3 [t_(9)_ = 0.66, p value = 0.52].

Pax6 is a well-known key regulator of glutamatergic neuronal differentiation, prompting us to investigate whether the expressions of Pax6 are inherited across generations as affected by VPA (Fig. 5B). Indeed, Pax6 expression was significantly increased in VPA-exposed F1 [t_(10)_ = 6.23, p value < 0.0001] as well as F3 generations [t_(8)_ = 3.75, p value = 0.0056] compared to their respective controls.

### VPA disrupts NMDA and AMPA receptor subtypes expression transgenerationally

The increased levels of NMDA and AMPA receptors in VPA-exposed rat offspring at 4 weeks old had been reported^33^. We conducted the Western blot analysis to evaluate whether NMDAR/AMPAR dysregulation are transmitted to the next generations even without subsequent VPA induction (Fig. 5C,D). The GluN1 and GluN2B protein levels in the cortex of mice were increased in both VPA-exposed F1 [GluN1: t_(12)_ = 3.38, p value = 0.0055, GluN2B: t_(14)_ = 4.29, p value = 0.0007] and F3 generations [GluN1: t_(18)_ = 4.28, p value = 0.0005, GluN2B: t_(12)_ = 3.81, p value = 0.0025]. Moreover, the AMPA receptor subtypes, GluR1 and GluR2, were highly expressed in both VPA-exposed F1 [GluR1: t_(14)_ = 3.20, p value = 0.0064, GluR2: t_(14)_ = 4.53, p value = 0.0005] and F3 generations [GluR1: t_(16)_ = 3.37, p value = 0.0039, GluR2: t_(14)_ = 4.57, p value = 0.0004].

## Discussion

The current study firstly elucidates that the effect of prenatal VPA exposure in offspring could be paternally transmitted from the first up to the third generation (F3). These effects were the autism-like behaviors and increased postsynaptic markers of excitatory neurons without affecting the number of pups born as well as their body weights across ages. Because the transmission experiments were performed with VPA-naïve female mice, the transmission is paternal and is not mediated by any abnormal maternal nurturing behaviors which the female ASD animals might have. Importantly, the teratogenic effects of VPA (crooked tail phenotype) appear only in F1 generation but not in subsequent generation showing the specificity of transgenerational transfer of autism-related behaviors. These results suggest that VPA-induced congenital effect is mediated by a mechanism other than transmissible epigenetic changes. The findings of this study present a new perspective in ASD susceptibility and heritability where autism can be inherited in subsequent generations after a pathologic environmental exposure in the first generation. Thus, this study supports the idea that epigenetic inheritance could play a role in the development and heritability of ASD.

The VPA-exposed animal models are widely studied models of ASD for their clinical relevance and validity^19^,^20^,^21^,^22^. A clinical entity called fetal valproate syndrome (FVS) was also named due to the effects of VPA in exposed human offspring^34^. Many researchers, including us have established that prenatal exposure of rats and mice to VPA at a specific time and dosage induces autistic-like phenotypes in the male offspring^26^,^33^,^35^,^36^,^37^,^38^,^39^. The VPA animal model displayed high connection and plasticity in the medial prefrontal cortex of their brain with increased NMDA/AMPA ratio^40^,^41^. There is also an increased expression of glutamatergic neuronal markers induced by the increased expression of Pax6 transcription factor^32^. These and other studies support the concept of excitatory/inhibitory imbalance as the pathologic mechanism underlying the VPA model of ASD. However, there is no published literature which describes a clinical evidence showing that VPA exposure in humans can induce a transgenerational inheritance of abnormal phenotypes and behaviors. Nevertheless, we conducted this study as a predictive validity to the increased prevalence of ASD in humans. This study gives us a hint that transgenerational inheritance may occur in humans although it has not yet been investigated. Additionally, there are numerous studies in literature supporting the involvement of environmental factors in transgenerational inheritance of disorders albeit the lack of such studies relating to ASD (reviewed in ref. 42). Hence, we addressed this possibility using the VPA animal model as a good example of an environmental cause of ASD to support the hypothesis that environment can play a crucial role in development and its effect in the germline can be transgenerationally inherited. Obviously, identifying ASD patients without evident genetic etiology and tracking the transgenerational transfer of abnormal behaviors in at least three generations would be a task requiring immense time and cost. Nevertheless, the results of this study encourage the investigation of epigenetic transgenerational inheritance of ASD in humans.

Environmental factors are closely associated with epigenetic changes in animal studies. Many environmental factors such as nutrition, drugs, endocrine disruptors and chemicals were reported to induce changes in the epigenetic status of cells^17^,^43^,^44^. Interestingly, maternal behaviors such as nursing behaviors and depression during pregnancy could affect the DNA methylation status of neurons^45^,^46^. Through various factors, the epigenetic changes in germ cells become imprinted and carried to the offspring^47^. In addition, the developmental effect of various environmental factors could disrupt the methylation of DNA in the developing embryo whereby affecting its development and carrying the trait to the succeeding generations^48^. For example, when pregnant rats were treated with endocrine disruptors (vinclozolin or methoxychlor) during the sex determination stage of embryos, decreased sperm count and fertility were observed in the male offspring which persisted transgenerationally up to the F4 generations^49^. Altered patterns of the DNA methylation architecture in the germline confirms the involvement of epigenetic effects in the development of offspring which were imprinted across multiple generations^49^. This implies that the intensive exposure to environmental factors especially affecting the germline makes a critical point that these changes are heritably bound to the offspring. The nature and extent of germline epigenetic changes induced by prenatal exposure to VPA should be determined in the future in relation with ASD phenotypes.

Previously, we elucidated that the histone deacetylase inhibitory activity of VPA affects the expression of various transcription factors such as Pax6 or methyl binding proteins such as MeCP2 in the brain of VPA-exposed rats which affected brain development leading to autistic-like phenotypes in the offspring^32^,^33^. Pax6 is involved in the regulation of development of glutamatergic neurons in the brain and MeCP2 is involved in the expression of genes through the control of DNA methylation-mediated chromatin accessibility. The expression of Pax6 is believed to be regulated by epigenetic factors such as Jarid1b and SNF5, a subunit of BAF chromatin remodeler complex^50^. Noting that Pax6 expression was sustained in succeeding generations after VPA exposure, as found in this study, gives us a hint that epigenetic changes and transmission are involved in the genetic expression of this transcription factor. Accordingly, it is reasonable to say that the epigenetic changes induced by VPA become imprinted in the germline of exposed offspring and are transmitted to the next generations. We are currently working on this essential aspect of the study and we are also determining whether the transgenerational epigenetic inheritance in the VPA-exposed offspring can as well occur through the maternal route, i.e. from mother to offspring despite that female animals did not show autistic-like effects in their behaviors^35^. In addition to the initial proposals and reports suggesting chemical-induced persistent and heritable epigenetic changes throughout the generation^48^,^51^,^52^, it is increasingly clear that the animal’s environment and stress level may modulate brain LTP and behaviors across multiple generations^53^,^54^. The remaining question would be the nature of the molecular biological mechanisms mediating these changes. It is a reasonable hypothesis that prenatal VPA exposure marks germline DNA in a way that it can modulate the trajectory of neuronal development related to the modulation of autism-related behaviors. Identifying the key modulators and their mechanisms would be a formidable but essential step to go further in this study.

Along with the transgenerational replicability of autistic-like symptoms in the VPA-exposed mice is the transgenerational replicability of neuronal protein expression involved in glutamatergic or excitatory neuronal development. We confirmed in the current study the heritability of these traits and suspect the involvement of epigenetic changes of Pax6 or its upstream regulators as the probable cause. Although more detailed studies are needed to confirm these insights, the preliminary steps presented in this study introduce a novel concept in ASD etiology in terms of environmental factor induction with transgenerational epigenetic inheritance. This concept has important implications in the increasing prevalence of ASD as the environmentally-induced epigenetic changes can be passed across generations. Moreover, this concept can also be applied in other diseases wherein the transgenerational consequences of high genetic susceptibility are also environmentally-rooted in the first place. Overall, this study offers more interest to the emerging field of transgenerational epigenetic inheritance and fills a new piece to the mysteries of ASD etiology.

## Methods

### Animals

Five-days-pregnant ICR mice (E5) were bought from Orient Bio Inc. They were kept in a facility equipped with a lighting system (lights on at 06:00 and off at 18:00), a temperature controller (22 ± 2 °C) and humidity maintenance (55 ± 5%). The animals were cared for and treated according to the Principles of Laboratory Animal Care (NIH publication No. 85–23, revised 1985). All animal experiments were approved by the institutional animal ethics review board of Konkuk University (KU12115). The number of animals used in experiments was minimized as possible. All tests were done from 10:00 to 16:00 o’clock in dedicated test rooms. At least six dams (individual F0 injected females) for control and another six dams for VPA-administered pregnant mothers comprised each batch of F1 to F3 generations. The F1 to F3 generations derived from VPA-injected F0 mothers are referred to as VPA-exposed, while those from saline-injected F0 mothers are identified as the control. Adult VPA-exposed F1 males were bred to newly-purchased naïve adult females to generate the VPA-exposed F2 while adult F2 males were similarly bred to generate the F3. Control groups were bred in the same manner across all generations. There were no inbreeding or sibling crosses that we generated for the subsequent offspring. Only male mice were utilized for all experiments.

### VPA prenatal injection

The sodium salt of VPA (Sigma, St. Louis, MO) was prepared in 0.9% saline at 50 mg/ml concentration and pH 7.4. VPA was injected to pregnant mice at 300 mg/kg of body weight on embryonic day 10. The control group received 0.9% saline as the vehicle.

### Open field test

The open field boxes are made of black Plexiglas (41.5 cm × 41.5 cm × 41.5 cm) with an overhead camera for automatic tracking of animal behaviors using the EthoVision System (Noldus, Wageningen, The Netherlands). The mice were carefully placed in the center of the open fields to measure their locomotor activity for 30 min using the video-tracking system. The locomotor activity was expressed as the total distance moved and the movement duration. The floors of the open field were cleaned with 70% ethanol in between trials.

### Social interaction test

The three-chamber social interaction test was originally adapted from Crawley’s group^55^, which we slightly modified. The three chambers are separated by a square door in the two inner walls connecting to the center area. Briefly, the test mouse was introduced in the center area to start habituation for 5 min and blocking access to the side compartments. The first test was conducted having a stranger mouse put inside an empty wire cage in one of the side chambers to measure the social interaction of the subject mouse without direct social contact. The other side was an empty wire cage. The next subsequent test introduced another stranger mouse in the previously empty wire cage to measure the preference for social novelty. Each test was performed for 10 min and the floor surfaces were wiped with 70% ethanol in between trials. The accumulated time spent in each compartment and the sociability or social preference indices were measured to quantify the social behavior of mice.

### Marble burying test

The marble burying test was performed with slight modifications from a previous report^56^. Animal cages were filled with clean corncob bedding (Anderson lab bedding, U.S.) up to 3 cm height and each mouse was put in individual cages for a 10-min habituation. Subjects were removed carefully from the cages momentarily while 20 glass marbles (15 mm diameter) were overlaid equidistantly in a 4 × 5 arrangement. Subjects were then returned to their designated test cages to explore the marbles for 20 min. The marbles buried in the bedding for >50% of their surface were counted and recorded. Twelve mice in each group were used for this analysis.

### Measurement of electroshock seizure threshold

The electric shock seizure threshold test was conducted in the 5-weeks old mice based on a previously established protocol^57^. Each subject received an electroshock stimulation for 1 s through ear clips connected to a current generator. An overt hind limb extension is considered a positive score for seizure. The electric current intensity is either decreased or increased depending on a positive or negative seizure score, respectively, to determine the electroshock seizure threshold. This ‘staircase’ method will identify the current level that provokes seizure in 50% of subjects called the convulsive current 50 (CC50)^58^. The results were calculated through the Litchfield–Wilcoxon II method^59^.

### Elevated Plus Maze (EPM)

The elevated plus maze assessed the anxiety-like behaviors in the control and VPA-exposed mice. The maze is made of four, black plexiglas arms shaped like a plus symbol with two open arms (67 × 7 cm) and two walled arms (67 × 7 × 17 cm) facing each other and connected by a neutral space in the center. The maze was mounted 55 cm above the floor and dimly illuminated (20 lux). The test started as the subject mouse was put on the neutral area facing an open arm and was allowed to explore all arms for 8 min. The time spent and the frequency of entry for each arm was automatically tracked by the camera-assisted EthoVision software. The data were presented as the percentage of time spent and frequency of entry in the open arms from the total time spent and entries in all arms.

### Western Blot Analysis

Isolated frontal cortical tissues were homogenized in ice-chilled Tris–HCl buffer (20 mM, pH 7.4). Protein concentrations were measured by the BCA assay. The loading control used was the relative amount of β-actin for each sample. Aliquots from the equalized protein samples were run in 10% SDS polyacrylamide gel electrophoresis for 120 min followed by the electrical transfer of proteins to nitrocellulose membranes for 90 min (Whatman, Germany). The blots were blocked with 5% nonfat dried milk at room temperature and incubated overnight at 4 °C with each specific primary antibody diluted at 1:1000 in 5% non-fat dried milk and then treated with horseradish peroxidase (HRP)-conjugated secondary antibody (Invitrogen, Carlsbad, CA, USA) for 2 h at room temperature. The protein bands were exposed to the LAS-3000 image detection system (Fuji, Tokyo, Japan) and enhanced by chemiluminescence (Amersham Biosciences, Piscataway, NJ, USA).

### Statistical analysis

The measures of behavioral experiments and the quantified protein blots were reflected in the box and whiskers plot (minimum, maximum, median, 25% quantiles, 75% quantiles). All data were presented as the mean and the standard error of mean (S.E.M). Statistical significance was analyzed using t-test and the significant p value was set at <0.05.

## Additional Information

**How to cite this article**: Choi, C. S. *et al.* The transgenerational inheritance of autism-like phenotypes in mice exposed to valproic acid during pregnancy. *Sci. Rep.* **6**, 36250; doi: 10.1038/srep36250 (2016).

**Publisher’s note:** Springer Nature remains neutral with regard to jurisdictional claims in published maps and institutional affiliations.
